# Comparison of antimicrobial resistance patterns of ESBL and non ESBL bacterial isolates among patients with secondary peritonitis at Bugando Medical Centre, Mwanza – Tanzania

**DOI:** 10.1186/s12873-016-0106-1

**Published:** 2016-10-21

**Authors:** Jeremiah Seni, Enock Sweya, Amri Mabewa, Stephen E. Mshana, Japhet M. Gilyoma

**Affiliations:** 1 Department of Microbiology and Immunology, Catholic University of Health and Allied Sciences, P.O. Box 1464, Mwanza, Tanzania; 2 Department of Surgery, Catholic University of Health and Allied Sciences, P.O. Box 1464, Mwanza, Tanzania; 3 Department of Surgery, Bugando Medical Centre, P.O. Box 1370, Mwanza, Tanzania

**Keywords:** ESBL, Secondary peritonitis, Mwanza, Tanzania

## Abstract

**Background:**

Secondary peritonitis is a common surgical emergence with deadly outcomes when not timely and promptly intervened. The emergence of Extended spectrum beta lactamase producing bacteria (ESBL) poses treatment challenge at Bugando Medical Centre (BMC); hence a need to evaluate the magnitude of ESBL so as to guide specific therapy.

**Methods:**

This was a cross sectional study conducted at BMC from May 2014 to April 2015 involving patients with secondary peritonitis. A questionnaire was used to collect patients’ information. Peritoneal aspirate sample was collected intra-operatively and processed using standard operating procedures to identify bacteria species and their susceptibility profiles.

**Results:**

The study involved 97 patients with the median age (IQR) of 32 (21–47) years, males were 62 (63.9 %) and about 27 (27.8 %) patients had co-morbid illnesses. The prevalence of ESBL among patients with secondary peritonitis was 23.7 % (23/97). Of 53 gram negative Enterobacteriaceae isolated, 47.2 % (25/53) were ESBL producers, with predominance of *Escherichia coli* 7 (28.0 %) and *Klebsiella spp* 5 (20.0 %). The ESBL isolates exhibited more resistance rates to trimethoprim sulfamethoxazole and ciprofloxacin compared to non ESBL isolates 96.0 % versus 60.7 %, *p* value = 0.003 and 16.0 % versus 0.0 %, *p* value = 0.043 respectively). All isolates were sensitive to meropenem.

**Conclusions:**

The prevalence of ESBL among patients with secondary peritonitis at BMC is high; with more resistance rates among ESBL compared to non ESBL isolates. There is a need for strengthen ESBL surveillance in this setting so as to guide specific therapy.

## Background

Peritonitis is the inflammation of peritoneum, the layer which enclose many organ in the abdomen [1]. Of the three types of peritonitis, secondary peritonitis has a major clinical impacts due to loss of integrity in hollow viscus resulting into perforation and is the most common form of peritonitis encountered in surgical practice causing significant morbidity and mortality when not timely and promptly intervened [1–4]. The most common causes of secondary peritonitis are perforated peptic ulcer disease, perforated small and large intestine, intestinal obstruction and many others [3–6]. The complications of secondary peritonitis are significant and include perforated viscus, hypovolemic shock, electrolyte imbalance, sepsis, multiorgan failure and deaths [1, 6].

The emergence of extended-spectrum beta lactamase (ESBL)-producing gram-negative infection in the past decade has made the management of gram-negative infections more difficult [7–9]; this in turn has necessitated a need to explore the magnitude of this problem across countries so as to confer specific therapy. It is well known that Gram negative bacteria of the family Enterobacteriaceae predominates in causing secondary peritonitis partly due to the close proximity and the possibility of translocation between peritoneum and bowel [1, 10, 11].

At Bugando Medical Centre (BMC), secondary peritonitis is the commonest indication for admission in the surgical wards; it is associated with significant workload, severe complications and deaths [6, 12, 13]. In our recent work at BMC, we delineated the etiology, treatment outcome and prognostic factors associated with secondary peritonitis [6]. To ensure rational antimicrobial therapy to patients with secondary peritonitis, this study was conducted to determine the proportion of bacteriologically confirmed patients with secondary peritonitis due to ESBL and their drug susceptibility profiles.

## Methods

### Study design, site and population

This was a cross sectional hospital based study conducted from May 2014 to April 2015 at BMC in Mwanza region Tanzania involving 97 patients with secondary peritonitis admitted at BMC who consented to participate in the study.

### Data collection and laboratory procedures

A pre-structured questionnaire was used to collect demographic and clinical data among consented patients. Approximately 5–8 ml of peritoneal aspirate sample was collected intra-operatively by the surgeon/resident and transported in the Catholic University of Health and Allied Sciences (CUHAS) multipurpose laboratory for processing within two hours using sterile swabs with Stuart’s transport media.

Gram stain for each sample was done followed by culture into Blood Agar, MacConkey Agar and Salmonella Shigella Agar (HI MEDIA, INDIA) and the plates incubated at 35–37 °C for 18–24 h. All gram negative bacteria were identified initially basing on lactose fermentation reaction on MacConkey agar. Then followed by in-house biochemical identification tests such as oxidase, citrate, urease, Triple sugar iron (TSI) and Sulphur indole motility (SIM) reactions [14]. Susceptibility testing was done using disc diffusion method basing on the Clinical Laboratory Standard Institute (CLSI) guideline [15]. The antibiotic discs tested included ampicillin (10 μg), trimethoprim/sulfamethoxazole (1.25/23.75 μg), chloramphenicol (30 μg), ciprofloxacin (5 μg), gentamicin (10 μg), amoxicillin/clavulanate (20/10 μg), ceftriaxone (30 μg), ceftazidime (30 μg), and meropemen (10 μg) (HI MEDIA, INDIA). The isolates were simultaneously screened for ESBL production in the same Muller Hinton Agar by using disc approximation method. Ceftazidime (30 μg) and Ceftriaxone (30 μg) discs were placed equidistant from the Amoxycillin/clavunate (20/10 μg) disc; enhanced zone of inhibition towards amoxycillin/clavunate disc were considered as positive result for ESBL production [7, 15, 16].

### Quality control

Standard laboratory procedures was strictly adhered. *Escherichia coli* ATCC 25922 and *Escherichia coli* ATCC 35218 and *Klebsiella pneumoniae* ATCC 700603 were used as negative and positive ESBL controls respectively for all laboratory procedures.

### Data management and analysis

Every participant had a unique identification number indicated on the questionnaire. The data and results were entered into a log book and later sorted and transferred into Microsoft excel for consistent checks and data cleaning. Data analysis was done using STATA version11.0 according to the objectives of the study. Results were presented into percentages/proportions for categorical variables, whereas median (interquartile range) was used to summarize the continuous variables and compared using Wilcoxon rank sum test. The chi-square (Fisher's exact where appropriate), *p*-value and odd ratio at 95 % confidence interval were used to test the significance association between ESBL-associated secondary peritonitis with deaths as well as comparison of resistance profiles between ESBL and non ESBL bacterial isolates.

## Results

Of the 97 patients with secondary peritonitis enrolled in this study, males were 62 (63.9 %). The median age (IQR) of participants was 32 (21–47) years, the minimum age was 5 years and the maximum age was 86 years. Majority of patients were primary school leavers 58 (59.8 %), with no employment 61 (62.9 %) and were from urban areas 51 (52.6 %). The study showed that about 13 (13.4 %) of enrolled patients were HIV seropositive and 27 (27.8 %) had co- morbidity (Table 1).

**Table 1:** Social demographic and clinical characteristics of the study participants

Parameter		Number	Percentages (%)
Sex	Female	35	36.1 %
	Male	62	63.9 %
Residence	Urban	51	52.60 %
	Rural	46	47.40 %
Education	Informal	14	14.40 %
	Primary	58	59.80 %
	Secondary	25	25.80 %
Occupation	Employed	20	20.60 %
	Unemployed	61	62.90 %
	Under 18	16	16.50 %
HIV	Negative	84	86.60 %
	Positive	13	13.40 %
Co-morbidity^a^	No	70	72.20 %
	Yes	27	27.80 %

^a^Puerperal sepsis (4), HIV (12), Severe anaemia (4), Hypertension (4), Tumor (1), Heart failure (1), Renal failure (1), HIV & Hypertension (1)

The proportion of patients with secondary peritonitis confirmed bacteriologically were 57.7 % (56/97); giving a total of 60 bacterial isolates (about 4 patients had double infections). The most common bacteria were *Escherichia coli*, 19 (31.7 %) and *Klebsiella spp*, 10 (16.7 %) (Table 2).

**Table 2:** Bacterial isolates from patient with secondary peritonitis at BMC

Bacteria	Frequency	Percentage (%)
*Escherichia coli*	19	31.7
*Klebsiella spp*	10	16.7
*Citrobacter spp*	9	15.0
*Enterobacter spp*	3	5.0
*Salmonella spp*	2	3.3
*Serratia marcescens*	1	1.7
Unidentified Enterobacteriaceae	7	11.6
Others^a^	9	15.0
Total	60	100.0

^a^ *Pseudomonas aeruginosa* (2), *Morganella morganii* (1), *Proteus vulgaris* (1), *Staphylococcus spp* (2), *Streptococcus spp* (2), and *Enterococcus spp* (1)

The prevalence of ESBL among patients with secondary peritonitis was 23.7 % (23/97). Of the Gram negative Enterobacteriaceae, 47.2 % (25/53) were ESBL producers; with majority being *Escherichia coli* 7 (28.0 %), and *Klebsiella spp* 5 (20.0 %) (Table 3).

**Table 3:** Proportions of ESBL among Enterobacteriaceae bacterial species

ESBL	Frequency	Percentage (%)
*Escherichia coli*	7	28.0
*Klebsiella spp*	5	20.0
*Citrobacter spp*	4	16.0
*Enterobacter spp*	3	12.0
*Salmonella spp*	1	4.0
*Serratia marcescens*	1	4.0
Unidentified Enterobacteriaceae	4	16.0
Total	25	100.0

There was a high resistance to ampicillin (100.0 %), amoxicillin-clavulanate (71.4–100.0 %) and trimethoprim-sulphamethoxazole (63.2–90.0 %); as opposed to low resistance rates to chloramphenicol (5.3–37.5 %), ciprofloxacin (0.0–15.8 %) and meropenem (0.0 %) (Table 4).

**Table 4:** Antimicrobial resistance profiles of the 53 Enterobacteriaceae bacterial species

Antimicrobial agent	Bacterial resistant patterns				
	*E.coli* (19)	*Klebsiella spp* (10)	*Citrobacter spp* (9)	*Unidentified* (7)	*Others (8)
	*n* (%)	*n* (%)	*n* (%)	*n* (%)	*n* (%)
AMP	19 (100.0)	10 (100.0)	9 (100)	7 (100)	8 (100.0)
C	1 (5.3)	1 (20.0)	2 (22)	2 (29)	3 (37.5)
AMC	18 (94.7)	9 (90.0)	8 (88.9)	5 (71.4)	8 (100.0)
CTR	7 (36.8)	4 (40.0)	4 (44.4)	4 (57.1)	5 (55.6)
CAZ	10 (52.6)	4 (40.0)	4 (44.4)	5 (71.4)	3 (55.6)
CO	12 (63.2)	9 (90.0)	8 (89)	5 (71.0)	7 (87.5)
GE	1 (5.3)	3 (30.0)	0 (0)	3 (43.0)	1 (25.0)
CIP	3 (15.8)	1 (10.0)	0 (0.0)	0 (0.0)	0 (0.0)
MEM	0 (0.0)	0 (0.0)	0 (0.0)	0 (0.0)	0 (0.0)

*AMP* ampicillin, *C* chloramphenicol, *AMC* amoxycillin-clavulanate, *CTR* ceftriaxone, *CAZ* ceftazidime, *CO* trimethoprim – sulfamethoxazole, *GE* gentamicin, *CIP* ciprofloxacin, *MEM* meropenem, *n* number of resistant bacterial species.

*Others: Enterobacter spp (3), Salmonella spp (2), Serratia marcescens (1), Morganella morganii (1), and Proteus vulgaris (1)

The ESBL isolates exhibited more resistance profiles in all antimicrobial agents compared to non ESBL isolates; the resistance rates to non beta lactam antibiotics like trimethoprim sulfamethoxazole and ciprofloxacin were significantly higher among ESBL isolates as opposed to non ESBL isolates (96.0 % versus 60.7 %, *p* value = 0.003 and 16.0 % versus 0.0 %, *p* value = 0.043 respectively) (Table 5).

**Table 5:** Comparison of antimicrobial resistance profiles of 25 ESBL and 28 non ESBL isolates

Antimicrobial agent	Bacterial resistant patterns		*p* value
	ESBL (*n* = 25)	Non ESBL (*n* = 28)	
	*n* (%)	*n* (%)	
AMP	25 (100.0)	28 (100.0)	-
C	7 (28.0)	2 (7.1)	0.067*
AMC	25 (100.0)	23 (82.1)	0.053*
CTR	25 (100.0)	0 (0.0)	<0.001*
CAZ	25 (100.0)	2 (7.1)	<0.001*
CO	24 (96.0)	17 (60.7)	0.003*
GE	4 (16.0)	4 (14.3)	1.000
CIP	4 (16.0)	0 (0.0)	0.043*
MEM	0 (0.0)	0 (0.0)	-

*ESBL* extended spectrum beta lactamase producer, *AMP* ampicillin, *C* chloramphenicol, *AMC* amoxycillin-clavulanate, *CTR* ceftriaxone, *CAZ* ceftazidime, *CO* trimethoprim – sulfamethoxazole, *GE* gentamicin, *CIP* ciprofloxacin, *MEM* meropenem, *n* number of resistant bacterial species

*Fisher’s exact

Although not significant, the proportions of patients who died was higher in ESBL-associated peritonitis group than non ESBL- associated secondary peritonitis group [21.7 % (5/23) versus 15.2 % (5/33) respectively, OR (95 % CI) = 1.56 (0.39–6.14); *p* value = 0.528).

The duration of hospital stay was relatively higher among patients with ESBL compared to those without ESBL [7 (6–16) days versus 6.5 (5–8) days; *p*-value 0.229)], despite the fact that no significant association was found.

There was no statistical association between ESBL associated secondary peritonitis with age, sex, residence, occupation, HIV serostatus and comorbid illness.

## Discussion

The magnitude of ESBL attributable infections is progressively growing all over the world with noticeable variation across countries and in difference clinical conditions [9]. This study showed high proportion of ESBL (47.2 %) among bacteria isolated from patients with secondary peritonitis. Similarly, higher prevalence rates between 70 and 86.5 % have been reported in Uganda, Ethiopia and India [17–19] as opposed to low prevalence rates of approximately 4.9–14.5 % reported in Netherland and Spain [20, 21]. Moreover, there is an increase in the magnitude of ESBL in Mwanza from 29.2 % in 2009; 35 % in 2014 and to 47.2 % in the present study in 2015 [7, 22]. The high prevalence in developing countries as opposed to developed countries may be due to lack of strict policies on the rational use of antimicrobial agents as well as lack of stringent measures on infection control and prevention strategies.

This study showed that the ESBL producing *E.coli* and *Klebsiella spp* were the leading organisms in patients with secondary peritonitis in approximately 28 and 20 % respectively. The findings are also similar to many other studies in different countries [8, 10, 11, 18, 19, 21]. The predominance of these species may be due to their presence as normal flora in the gastrointestinal tract and the possibility of traversing to the peritoneal cavity due to anatomic close proximity.

Similar to other studies, majority of ESBL isolates showed high resistance to commonly used antimicrobial agents such as trimethoprim sulfamethoxazole (>80 %) as opposed to less commonly used antimicrobial agents such as ciprofloxacin and imipenem [10, 18, 19]. Moreover, the resistance rates in various antimicrobial agents were shown to be higher among ESBL isolates as opposed to non ESBL isolates reiterating the need to have laboratory guided screening strategy in this setting, so as to timely identify patients with ESBL associated infections and thus, provide specific therapy.

Despite the fact that there was no statistical significance, the study showed that the proportions of patients who died was more in the ESBL-associated secondary peritonitis group than non ESBL- associated secondary peritonitis group calling for a large prospective study with large sample size to ascertain these findings. High mortality in patients with multi drug resistance bacterial infections have also being reported in in other studies in Tanzania, Uganda and Spain [11, 19, 23]. Therefore these findings, emphasize on the negative impact of multi-drug resistant bacteria and hence a need for continuous antimicrobial resistance surveillance so as to guide specific therapy and better outcome among patients.

### Study limitations

Anaerobic culture was not done in the present study due to lack of facilities for this technique; this may underestimate the actual number of bacteria involved in secondary peritonitis.

## Conclusions

The prevalence of ESBL among patients with secondary peritonitis at BMC is high (23.7 %). There is also high proportion of Gram negative Enterobacteriaceae (47.2 %) exhibiting ESBL phenotype; with majority of these isolates being *E.coli* and *Klebsiella spp*. Moreover, there is a growing problem of ESBL in Mwanza - Tanzania for the past 6 years. Although not statistically significant, patients with ESBL associated secondary peritonitis were more likely to die as opposed to those without ESBL.

There is a need to have a continuous ESBL surveillance at BMC so as to guide specific antimicrobial therapy. A study to evaluate the impact of anaerobic bacteria should be carried out in this setting, so as to assess their impact among patients with secondary peritonitis.

## Abbreviations

ATCC: American type culture collection
BMC: Bugando Medical Centre
CLSI: Clinical Laboratory Standard Institute
CUHAS: Catholic University of Health and Allied Sciences
ESBL: Extended spectrum beta lactamase producing bacteria
SIM: Sulphur indole motility
TSI: Triple sugar iron
